# The Economics of Tuberculosis

**Published:** 1906-10-13

**Authors:** 


### The Hospital

A Journal of

The Medical Sciences and Hospital Administration.

Vol. XLI.—No. 1,047. SATURDAY, OCTOBER 13, 1906.

### THE ECONOMICS OF TUBERCULOSIS.

Since the discovery of the tubercle bacillus by Professor Koch tuberculosis in its pathological relationships has become a comparatively simple and commonplace problem. On the other hand, there have arisen in connection with the disease large social and economic questions to which it is of the first importance careful heed should be given. In another column we present to our readers an essay dealing with these questions, and hope in this way to contribute to the education of public opinion on a subject which concerns both the national welfare and the wise and economic use of public money. The writer, Dr. Chas. Reinhardt, is well entitled to speak with authority on the subjects of which he writes. He has given to these subjects long and earnest attention, and has enjoyed practical opportunities of studying various methods of dealing with them. Hence, whilst he holds decided views and has no hesitation in expressing his opinions, these are not the outcome of doctrinaire authority, but are based on actual experience of the several plans which have been advocated as a solution of existing difficulties. The contribution which he makes to the subject is at least the result of first-hand knowledge, and as such is entitled to the most serious consideration.

The social and economic problem presented by tuberculosis is in its essential facts contained in a few brief statements. The first of these is that the disease is responsible for sixty thousand deaths, to take only England and Wales, every year. Following this is the reflection that the majority of these deaths occur in young adults, and that in this way the national efficiency is diminished and large charges are made upon the public purse. The picture drawn by Dr. Reinhardt of the course of events set into operation when phthisis pulmonalis attacks the bread-winner of the family owes its dramatic force, not to exaggeration, but to a life-like presentation of the facts and to the frequency with which these are repeated. It is a heart-breaking record, and for the great majority of the victims of the disease it is in existing circumstances as inevitable as fate. Were all this written of a disease from which escape was impossible and in which no prospect of cure existed, human ingenuity and industry might still be stimulated to endeavour to find a way out. The courageous, at all events, would not readily give up the struggle. But the position in reference to phthisis pulmonalis is exactly the opposite of the one just suggested. So far from being beyond control, it is without question a preventable disease, and, at least when taken in the early stages, it is, in the vast majority of cases, curable. Here, then, is the sharp and almost bewildering contrast. A disease which in our own country is responsible for one death in every six, which is a fruitful source of pauperism and poverty, and which costs the nation many millions every year, is one which is capable of being altogether suppressed, and which, in the individual, can almost invariably be cured if proper measures are prompely applied. Though it is known the plague may be stayed, its progress as a matter of fact continues.

Now it is quite certain that the existing measures for dealing with tuberculosis are not only inadequate, but they are based upon a method which will necessarily leave them permanently inadequate. If the plague can only be suppressed by the erection of sanatoria costing from £500 to £1,000 per bed, it must be quietly accepted that the desired end is not within the range of practical politics. Neither private benevolence nor public funds can be found to meet the necessary charges. The cure of cases of phthisis pulmonalis occurring among the working classes must be abandoned as frankly impossible—a position which also involves the continued dissemination of the disease throughout the community. In short, however desirable the abolition of tuberculosis is allowed to be, we must, on the above supposition, regretfully come to the conclusion that we are not able to afford the expense.

Now Dr. Reinhardt's solution of the sanatorium difficulty is strong just where strength is required. He protests that palatial buildings are entirely unnecessary. Even more, they are not only expensive, they are also inefficient. On the other hand, the "chalet" system permits the organisation of an efficient sanatorium at an outside expense of £100 per bed, and also satisfies the conditions necessary for the successful treatment of tuberculosis more fully than is done in a large hospital-like building. All this, it will be observed, is not merely in the domain of academic possibility. The plan rests on experience and on financial and therapeutic success. And valuable and important as it is to the individual patient, it touches also the larger question of the relationship of the national responsibility to the consumptive poor. That is a question which sooner or later will have to be faced. It carries grave moral and economic responsibilities, and those who attempt its solution must be equipped in both these respects. Dr. Reinhardt's essay is a help to this end, as well as a suggested solution of an immediate difficulty.

